# Perniosis (chilblains) masquerading as CA-MRSA: a case report

**DOI:** 10.1186/1757-1626-2-6500

**Published:** 2009-05-29

**Authors:** Keith D Bohman, Thomas J Papadimos, Lorie D Gottwald, Zhixing K Pan

**Affiliations:** 1 Department of Pathology, University of Toledo, College of Medicine3000 Arlington Avenue, Toledo, OH 43614USA; 2 Department of Medical Microbiology and Immunology, University of Toledo, College of Medicine3000 Arlington Avenue, Toledo, OH 43614USA; 3 Department of Anesthesiology, University of Toledo, College of Medicine3000 Arlington Avenue, Toledo, OH 43614USA; 4 Department of Dermatology, University of Toledo, College of Medicine3000 Arlington Avenue, Toledo, OH 43614USA

## Abstract

Perniosis (chilblains) is a vasospastic, inflammatory disease that occurs when the skin is subjected to cold above the freezing point, under damp conditions. Erythematous (violaceous) blisters, ulcerations or pustules that sit on an edematous base, accompanied by pain, burning or itching, are usually evident. To the inexperienced clinician it may resemble community-associated methicillin-resistant *Staphylococcus aureus* and could lead to inappropriate treatment. Here we report such a case.

## Introduction

Perniosis (chilblains) is a vasospastic, inflammatory disorder that occurs when the skin of individuals is unprotected and exposed to a nonfreezing, cold and damp environment [1]. The discoloration of the extremities and any accompanying lesions may resemble methicillin-resistant *Staphylococcus aureus* (CA-MRSA) [2]. In this era of heightened awareness regarding CA-MRSA, the unfamiliarity of medical practitioners with perniosis may lead to inappropriate hospital admissions and therapies [3]. Here we report a case of presumed bacterial skin infection of the feet treated as CA-MRSA when, in fact, it was perniosis.

## Case presentation

A 23-year-old gravida 0, para 0, Caucasian female graduate student weighing 55 kg and 160 cm in height presented to her primary care physician with discolored digits (1 through 3) on both of her feet. She also complained that her toes were painful, blistered, and intermittently pruritic. She stated her symptoms had begun the day after receiving a pedicure before going out to a university event that same evening. The patient's vital signs were blood pressure 120/62 mm Hg, heart rate of 70 beats per minute, and respirations 14 per minute. Physical examination of her heart and lungs was unremarkable. Her medical and surgical histories were negative. She was a non-smoker and consumed less than 6 ounces of alcohol per week. She had no allergies. The primary care physician prescribed cephalexin 500 mg by mouth daily for 10 days for a presumed bacterial infection.

**Figure 1.**
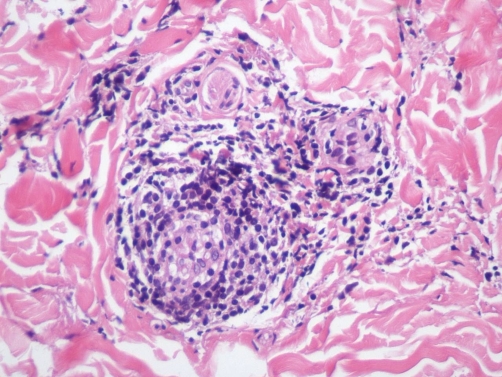
Hematoxylin and Eosin (40x). This high power view shows perivascular lymphocytes with intravascular fibrin indicative of early thrombus formation in our patient.

**Figure 2.**
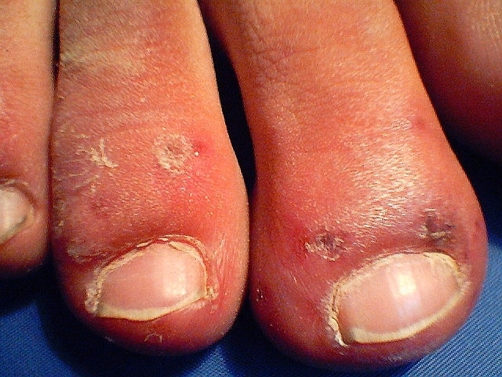
Perniosis of the toes, example that could be confusing.

Ten days later she still complained of discoloration, blisters and pain, and decided to visit an Emergency Department (ED) for a second opinion. The ED physician diagnosed CA-MRSA based on the physical presentations of the lesions and the increased incidence of MRSA in the community. Trimethoprim-sulfamethoxazole double strength (160 mg/800 mg) orally every 12 hours for 10 days was prescribed. The symptoms and signs persisted and the patient arranged to see a dermatologist, who performed a biopsy of the third digit (left foot). The dermatologist declined to prescribe further treatment until the biopsy returned. The biopsy revealed a histological diagnosis of perniosis (see Figures 1,2). On further inquiry by the dermatologist, the patient stated that the evening after her pedicure she had spent several hours walking outdoors before the aforementioned university event. She had worn open-toed shoes and the wind chill factor that evening had gone as low as 0° F.

The patient was counseled to keep her feet warm and avoid damp conditions. No other medications were administered and the symptoms resolved three weeks later (mid-April) with conservative care and the return of warmer ambient temperatures.

## Discussion

The prevalence of perniosis in the United States is unknown. The lesions may be single or multiple, erythematous and usually involve the hand, feet or face (fingers, toes, nose, or earlobes) [1]. The lesions have also been found on the hips [4] and thighs [5] of patients. The lesions of the toes are similar to MRSA and physicians' lack of familiarity with perniosis may lead to inappropriate hospital admissions with expensive evaluations and hazardous treatments [3].

Differential diagnosis includes systemic lupus erythematosis, cryopathy or connective tissue disease (with loss of seasonal patterns), polycythemia vera, septic emboli, Raynaud's syndrome, atheromatous embolization, pernio sarcoid, erythromelalgia, vasculitis, peripheral artery disease, and purple toe syndrome from anticoagulant therapy [1,3], and now CA-MRSA can be added to this list.

The foremost treatment option requires the use of appropriate insulated clothing, avoiding immobility, warm housing, proper exercise and nutrition, and avoiding skin exposure to damp, cold environments [1,3]. Furthermore, nicotine use should be discouraged and alcohol intake limited [1]. Medication may be used as an adjunct to the above interventions, and includes nifedipine or amlodipine [1]. These medications increase digital blood flow, reduce healing time, and lessen pain and irritation [6-9]. They are prescribed until warm weather returns and restarted in the autumn as prophylaxis [1]. Ultraviolet light, topical corticosteroids, calcium preparations, intramuscular vitamin injections, and vasodilators have been used in the past, but are probably ineffectual [1,3].

## Conclusions

In this report the necessity of a complete history is evident. The temporal relation of the patient's pedicure may have led the initial physicians' evaluation astray, allowing the cold exposure to be overlooked. This aspect of the patient's interview cannot be overemphasized. Additionally, the epidemic of CA-MRSA occurring in the United States may have caused the clinicians to focus on this organism because of its resistance and virulence, possibly to the exclusion of other diagnoses. Nonetheless, we encourage our colleagues to continue to be vigilant in regard to CA-MRSA, but we further emphasize the need for physicians in the primary care setting to understand and recognize cold weather injuries.
